# Does Emotional Intelligence at medical school admission predict future licensing examination performance?

**DOI:** 10.36834/cmej.67884

**Published:** 2020-03-16

**Authors:** Timothy J. Wood, Susan Humphrey-Murto, Geneviève Moineau, Melissa Forgie, Derek Puddester, John J. Leddy

**Affiliations:** 1 Department of Innovation in Medical Education,University of Ottawa, Ontario, Canada; 2 Department of Medicine, University of Ottawa, Ontario, Canada; 3 Department of Pediatrics, University of Ottawa, Ontario, Canada; 4 Department of Psychiatry, University of Ottawa, Ontario, Canada; 5 Department of Cellular and Molecular Medicine, University of Ottawa, Ontario, Canada

## Abstract

**Background:**

Medical school admissions committees are seeking alternatives to traditional academic measures when selecting students; one potential measure being emotional intelligence (EI). If EI is to be used as an admissions criterion, it should predict future performance. The purpose of this study is to determine if EI scores at admissions predicts performance on a medical licensure examination

**Methods:**

All medical school applicants to the University of Ottawa in 2006 and 2007 were invited to complete the Mayer-Salovey-Caruso Emotional Intelligence Test (MSCEIT v2.0) after their interview. Students were tracked through medical school into licensure and EI scores were correlated to their scores on the Medical Council of Canada Qualifying Examination (MCCQE) attempted between 2010 and 2014.

**Results:**

The correlation between the MSCEIT and the MCCQE Part I was *r* (200) = .01 *p* =. 90 The covariates of age and gender accounted for a significant amount of variance in MCCQE Part I scores (*R*^2^ = .10, *p*<.001, n=202) but the addition of the MSCEIT scores was not statistically significant (*R*^2^ change = .002, *p*=.56). The correlation between the MSCEIT and the MCCQE Part II was *r*(197) = .06, *p* = .41. The covariates of age and gender accounted for some variance in MCCQE Part II scores (*R*^2^ = .05, *p* = .007, n=199) but the addition of the MSCEIT did not (*R*^2^ change = .002 *p* =.55).

**Conclusion:**

The low correlations between EI and licensure scores replicates other studies that have found weak correlations between EI scores and tests administered at admissions and during medical school. These results suggest caution if one were to use EI as part of their admissions process.

## Abstract (fr)

**Contexte:**

Les comités d’admission des facultés de médecine sont à la recherche d’alternatives aux mesures académiques traditionnelles lors de la sélection des étudiants; une mesure potentielle étant l’intelligence émotionnelle (IÉ). Si l’IÉ doit être utilisée comme critère d’admission, elle devrait pouvoir prédire la performance future. L’objectif de cette étude est de déterminer si les résultats de l’IÉ lors des admissions prédisent la performancelors d’un examen de certification.

**Méthodes:**

Tous les candidats à la faculté de médecine de l’Université d’Ottawa en 2006 et en 2007 ont été invités à remplir le test d’intelligence émotionnelle Mayer-Salovey-Caruso (MSCEIT v2.0) après leur entrevue. Les étudiants ont été suivis tout au long de leurs études en médecine jusqu’à leur début de pratique et les résultats de l’IÉ ont été corrélés à leurs résultats lors de l’examen d’aptitude du Conseil médical du Canada (EACMC) tentéentre 2010 et 2014.

**Résultats:**

La corrélation entre le MSCEIT et la partie I de l’EACMC était de *r* (200) = 0,01 *p* = 0,90. Les covariables de l’âge et du genre comptaient pour une partie importante de la variance dans les résultats de la partie I de l’EACMC (*R*^2^ = 0,10, *p* < 0,001, n = 202), mais l’ajout des résultats du MSCEIT n’était pas statistiquement significatif (changement de *R*^2^ = 0,002, *p* = 0,56). La corrélation entre le MSCEIT et la partie II de l’EACMC était de *r* (197) = 0,06 *p* = 0,41. Les covariables de l’âge et du genre comptaient pour une certaine variance dans les résultats de la partie II de l’EACMC (*R*^2^ = 0,05, *p* = 0,007, n = 199), mais l’ajout des résultats du MSCEIT ne comptait pas (changement de *R*^2^ = 0,002, *p* = 0,55).

**Conclusion:**

Les faibles corrélations relevées entre les résultats de l’IÉ et la certificationconfirment les conclusionsd’autres études qui ont obtenu de faibles corrélations entre les résultats de l’IÉ et les tests donnés aux admissions et à la faculté de médecine. Ces résultats appellent à la prudence si quelqu’un devait utiliser l’IÉ dans son processus d’admission.

## Introduction

Medical schools often base student admission on a combination of academic and non-academic measures.^1^ Grade point average or standardized tests like the Medical College Admission Test (MCAT) assess academic measures, which are typically related to academic ability, processing of information, reasoning comprehension, and decision-making. These academic measures have been found to be predictive of scores on licensing examinations, internship ratings, and career progression.^1^^-^^5^Researchers have found non-academic measures to be more challenging to define and measure. They are related to traits like interpersonal communication, empathy, teamwork, self-awareness^1^^,^^6^ and medical schools usually use tools like autobiographical sketches, reference letters and interviews to evaluate them. Measures based on these tools have been found to have, at best, only a moderate relationship to future performance in medical school;^2^^,^^5^^,^^7^^,^^8^ a finding that has contributed to the adoption of alternate measures that capture non-academic ability like the multiple mini-interview (MMI)^7^ or the Computer-based Assessment for Sampling Personal characteristics (CASPer).^9^

In addition to test formats like the MMI and CASPer, another construct that medical schools could use as a non-academic measure is emotional intelligence (EI). EI is related to the capacity to monitor one’s own and others’ emotions, to discriminate among emotions, and to use emotions to guide thinking and actions especially around interpersonal and communication skills.^10^ These are important characteristics for a physician because understanding patients’ emotions and controlling one’s own emotions are essential to maintaining effective doctor–patient relationships and to working successfully in teams.

EI has garnered significant attention in medicine. Systematic reviews reveal that higher EI scores contribute to improved doctor–patient relationships, increased empathy, improved teamwork and communication skills, as well as better stress management, organizational commitment, and leadership skills.^11^ In addition, factors that may increase EI in medical students have been identified and include EI and empathy training later in undergraduate education, emphasizing empathetic communication styles, and gender.^12^ The findings from these reviews suggest that incorporating EI as a non-academic measure during the medical school admission process could have value.

Findings that have considered EI during medical school admissions have not been as conclusive as the above studies suggestWhen compared to scores on other traditional admissions measures (e.g., GPA, interview, autobiographical sketch) there does not appear to be a predictive relationship.^13^^-^^15^ Similar results involving EI scores and the MCAT, American College Test (ACT), GPA and MMI have been reported.^16^^-^^19^Carrothers et al.,^17^ however, did find a significant correlation between EI and an interview score. When compared to other tests or performance measures conducted during medical school and residency, findings have been mixed. Several studies have reported significant positive relationships between EI and other knowledge and clinical tests;^16^^,^^20^^-^^23^in contrast, other studies found no correlation between EI and other medical school measures^18^^,^^24^^,^^25^ or between EI and the performance of academically underperformingfirst year medical students.^26^

It is possible that the discrepancy between studies is related to how these studies measure EI. Researchers distinguish between two methods of measuring EI: Trait EI and Ability EI.^10^^,^^11^^,^^27^^,^^28^ Trait EI assumes that EI is related to individual personality traits and uses self-reported measures. Common examples of trait EI tests include the Trait Emotional Intelligence Questionnaire (TEIQue) and the Bar-On Emotional Quotient Inventory (EQi). Ability EI assumes that EI is a cognitive ability related to reasoning and problem solving about emotions. The most commonly used and widely studied example of an ability EI test is the MSCEIT v2.0. Correlations between trait EI scores and MSCEIT scores tend to be low.^27^^,^^28^There is also a second ability measure of EI that uses a variation of a situational judgment test. Examples of these tests include the Situational Test of Emotion Management (STEM) and the Situational Test of Emotional Understanding (STEU). Research has found some associations between the STEM, STEU and the MSCEIT and low correlations with other trait EI tests.^27^ Unfortunately, all of these tests have been used to make comparisons between EI and other medical school tests and consistent patterns have not been found, even when using the same measure of EI.

Despite these discrepant findings, EI continues to be of interest to health professions educators with recent papers exploring its role in workplace assessment,^29^ whether it can be taught,^30^^,^^31^ and in leadership training.^32^ Given this continued interest, the possibility of using EI as an admissions tool remains pertinent. One marker of success for any medical student, and one that that has not been well studied, is the degree to which EI could predict scores on a medical licensing examination. In one example, EI scores for surgery residents were compared to scores on the United States Medical Licensing Examination (USMLE) Steps 1 - 3 and the American Board of Surgery In-Training Examination (ABSITE).^33^Findings showed that EI scores were positively correlated with USMLE Step 2 and Step 3 scores but not USMLE Step 1 or ABSITE scores. Gardner and Dunkin report similar non-significant correlations between EI and USMLE scores for surgery residents.^34^ The participants in both of these studies were residents who had attempted the EI after medical school; therefore, generalizing the findings to undergraduate admissions is risky.

The purpose of our study was to explore the use of EI as a measure of non-academic skills at medical school admissions by comparing EI scores to performance on a medical licensing examination. It is expected that EI scores, which are thought to capture the perception and management of emotions, should be poorly related to aspects of a licensure examination that measure basic medical knowledge and clinical decision-making. In spite of inconsistent results comparing EI to other medical school measures, we expected that EI scores would be correlatedto aspects of a licensure examination that measure patient interaction skills because these skills are related to building rapport and trust with a patient and should be influenced by the ability to perceive and manage emotions.

## Methods

### Admissions process

The University of Ottawa Faculty of Medicine annually selects approximately 165 applicants from a large pool of approximately 4,000 applicants for admission to its M.D. program. This process was described in a previous paper^15^ but, in summary, students are first screened on the basis of the weighted GPA (wGPA) and an autobiographical sketch score. Successful applicants then participate in a semi-structured interview with the final decision based on the results of that interview combined with the wGPA.

### Participants

All applicants in 2006 and 2007 who were offered an interview were invited to participate in the study. Those who agreed to participate completed the MSCEIT v2.0 in English or French immediately after their admissions interview. A proctor delivered the MSCEIT v2.0 and an outsider vendor, Multi-Health Systems Inc., scored the tests.

### Ethics

The Ottawa Health Sciences Network Research Ethics Board approved the following research design.

### MSCEIT v2.0

There are multiple tools designed to assess EI, but the MSCEIT v2.0 was used because it focuses on the ability to perceive emotions in others and to manage one’s own emotions rather than the knowledge of emotions typically tested in trait-based, self-reported rating scales like the TEIQue or the EQi. The MSCEIT has also been shown to have good psychometric properties.^35^^,^^36^

The MSCEIT v2.0 consists of 141 items and each item is scored by a general consensus method in which a respondent’s answer is scored according to the proportion of the reference sample of the general adult population (n=5000) and/or the proportion of emotion experts that endorsed the same MSCEIT answer. Results from this consensus scoring are compiled to generate a total EI score with a mean of 100 and a standard deviation of 15.^36^ As reported in a previous study,^15^ Cronbach’s Alpha for the cohorts used in this study was .86 and .87.

### MCC Qualifying Examination

The Medical Council of Canada (MCC) administers a high-stakes examination called the MCC Qualifying Examination (MCCQE). This examination comprises two different examination formats, the MCCQE Part I and the MCCQE Part II. The purpose of the MCCQE Part I is to assess basic medical knowledge and clinical decision-making at the level of a graduating medical student. At the time of the study, the MCCQE Part I had two components: a multi-stage computer-adaptive multiple-choice question examination and a form-based set of key feature short answer questions. Total scores on any given administration were standardized to have a fixed cut-score of 390 and a SD of 100. The MCCQE Part II is a ten-station Objective Structured Clinical Examination (OSCE) designed to measure clinical skills related to history taking, data acquisition and patient interaction skills. Only the total score was used in this study because MCC expresses caution when reporting sub-scores. The reasoning is that sub-scores like patient interaction, have fewer measurement points and therefore have less precision. In addition, sub-scores are also not linked across administrations making comparisons from year to year difficult. It should be noted however, that the total score still reflects the contribution of communication and patient interaction skills. Examinees were scored by physician examiners who used a combination of station-specific checklists and global ratings. At the time of the study, total scores on the MCCQE Part II were standardized to have a mean of 500 and a standard deviation of 100 To be eligible to attempt the MCCQE Part II, examinees must have passed the MCCQE Part I and have one year of clinical post-graduate training. Successful completion of both parts of the Qualifying Examination entitles a candidate to become a Licentiate of the Medical Council of Canada, which is a prerequisite to medical licensure in Canada.

## Analysis

Due to confidentiality reasons, we were not able to link the MCCQE Part I exam data to the MCCQE Part II exam data, and therefore separate analyses were conducted for each examination. Descriptive statistics for the total score on the MSCEIT v2.0 and the MCCQE Part I and Part II were determined for the combined cohort and then repeated for each cohort (2006 and 2007). In order to compare participants’ MSCEIT scores with their MCCQE Part I and MCCQE Part II scores, the correlation between the MSCEIT and examination scores was calculated. Correlations, however, can be influenced by other variables that are related to the dependent variable. For example, age and gender have been found to correlate with EI scores^11^^,^^12^ and with USMLE scores.^37^ Therefore, the possibility exists that they could influence a correlation between EI and MCCQE scores. To ensure we had an accurate measure of the relationship between the measures, we also conducted a hierarchical linear regression in which age and gender were used as covariates with the MSCEIT score treated as an independent variable and the respective MCC score as the dependent variable. The advantage of this analysis is that it will remove any variance associated with Age and Gender so that the resulting comparison between the MSCEIT V2.0 score and the MCC score will be more precise.

## Results

**Figure 1:**
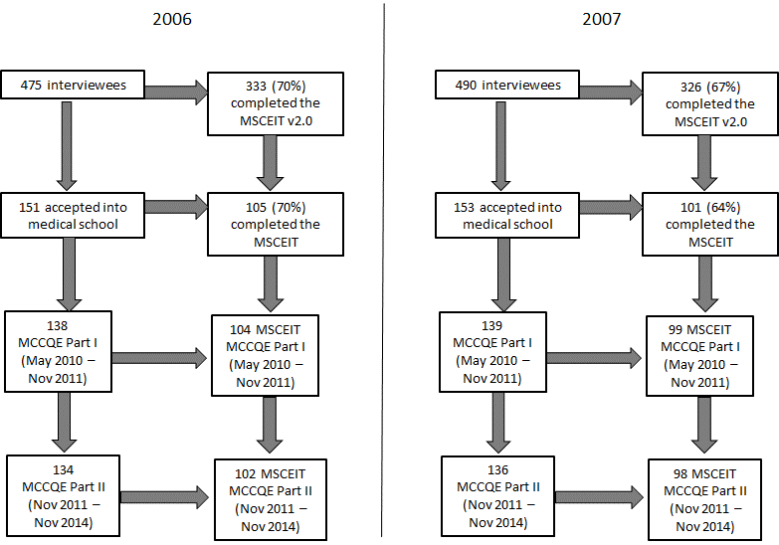
Flowchart of the number of students accepted into medical school, completed the MSCEIT and attempted the MCCQE Part 1 and MCCQE Part II

Figure 1 displays a flowchart of the number of participants at each level of the study. For the 2006 cohort, 333/475 (70%) interviewees completed the MSCEIT v2.0. Of this number, 151 were accepted into our medical school, 105 of who had completed the MSCEIT 2.0. For the 2007 cohort, 326/490 (67%) interviewees completed the MSCEIT v 2.0. A total of 153 were accepted into our medical school, 101 of who had completed the MSCEIT 2.0.

### MCCQE Part I

Between May 2010 and May 2012, a total of 138/151 (91%) students from the 2006 cohort attempted the MCCQE Part I. Of these, 104 had MSCEIT v2.0 scores. For 2007, 139/153 (91%) students had MCCQE Part I scores with 99 having MSCEIT v2.0 scores. Scores for one participant in the 2007 cohort were removed because we did not have age data. It is not clear why the match to the MCC databases was not higher and could be related to name changes, leave of absences, or delays in writing the examination.

For the 2006 cohort, the mean age was 23.65 years (+/- 2.96 years). A total of 64 of the participants were male and 40 were female. For the 2007 cohort the mean age was 23.17 years (+/- 3.31years). A total of 60 of the participants were male and 38 were female. As shown in Table 1, the mean total MSCEIT v2.0 score was 98 (*SD* = 10) for the 2006 cohort and 96 (*SD* = 12) for the 2007 cohort with a combined mean of 97 (*SD* = 11). For the most part, these scores fall within the usual range of the MSCEIT (i.e., +/- 1 *SD* of the mean). The mean MCCQE Part I score was 511 (*SD* = 72) for the 2006 cohort, 537 (*SD* = 63) for the 2007 cohort and 524 (*SD* = 69) for the combined cohorts.

**Table 1:** Descriptive statistics for the MSCEIT v2.0 and MCCQE Part I

	2006						2007						Combined					
	n	M	SD	Mdn	Min	Max	n	M	SD	Mdn	Min	Max	n	M	SD	Mdn	Min	Max
MSCEIT^a^	104	98	10	99	65	118	98	96	12	97	41	117	202	97	11	98	41	118
MCCQE Part 1^b^	104	511	72	515	325	695	98	537	63	538	399	737	202	524	69	525	325	737

Abbreviations: MSCEIT = Mayer-Salovey-Caruso Emotional Intelligence Test; MCCQE Part I = Medical Council of Canada Qualifying Examination Part I.

a = Mean score = 100, SD = 15

b = reference cut-score = 390, SD = 100

**Figure 2:**
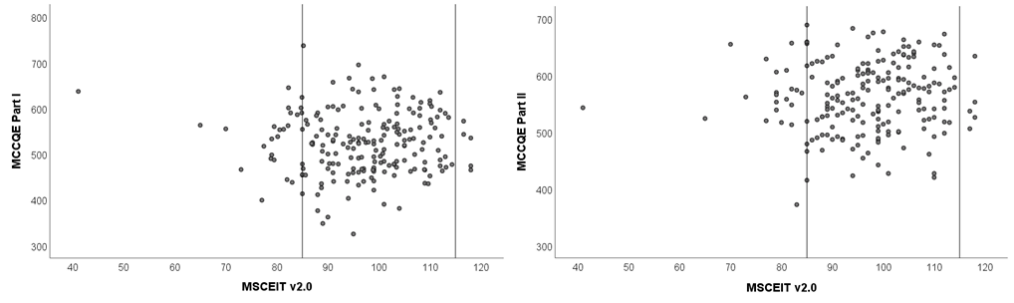
Scatterplots for the MSCEIT v2.0 score vs. MCCQE Part I and Part II

Note. Vertical lines in each scatterplot represent +/- 1 standard deviation from the MSCEIT mean

For the combined cohorts, the relationship between the MSCEIT and the MCCQE Part I can be seen in Figure 2. As shown, there does not appear to be a strong relationship between the scores on the two measures. Even participants with extremely low or extremely high MSCEIT scores (i.e., more than 1 standard deviation from the mean) have a range in MCC Scores. The correlation between measures was *r*(200) = .01, P = .90, 95% CI [-.13, .15]. When the covariates of age and gender are included, they account for a statistically significant amount of variance in MCCQE Part I scores (*F*(2,199) = 10.87, *R*^2^ = .10, *p*<.001) but the addition of the MSCEIT was not statistically significant (*F*(1,198)=.333, *R*^2^ = .10, *R*^2^ change = .002 *p*=.56). For 2006, the correlation between the MSCEIT scores and the MCCQE Part I was *r*(102) = .11, P = .25, 95% CI [-.08, .30]. The covariates of age and gender accounted for a significant amount of variance (*F*(2,101) = 5.88, *R*^2^ = .10, *p* =.004) however, there was a non-significant increase in *R*^2^ of .02 when the MSCEIT V2.0 was considered (*F*(1,100) = 2.15, R^2^ = .12, *p* =.15). Similarly, for the 2007 cohort, the correlation was *r*(97) = -.08, P = .46, 95% CI [-.27, .12] with age and gender accounting for significant amount of variance in scores (*F*(2,95) = 5.13, *R*^2^ = .10, *p* =.008 but a non-significant increase in *R*^2^ of .01 when the MSCEIT score was considered (*F*(1,94)=.54, *R*^2^ = .10, *p*=.47).

### MCCQE Part II

Between May 2011 and November 2014, a total of 134 people from the 2006 cohort attempted the MCCQE Part II. Of these, 102 had MSCEIT v2.0 scores. For 2007, 136 people had attempted the MCCQE Part II with 98 having MSCEIT v2.0 scores. As shown in Table 2, the mean MSCEIT v2.0 at interview was 98 (*SD* = 10) for the 2006 cohort and 96 (*SD* = 12) for the 2007 cohort with a combined mean of 97 (*SD* = 11). The mean MCCQE Part II score was 554 (*SD* = 64) for the 2006 cohort, 571 (*SD* = 57) for the 2007 cohort and 562 (*SD* = 61) for the combined totals.

**Table 2:** Descriptive statistics for the MSCEIT v2.0 and MCCQE Part II

	2006						2007						combined					
	n	M	SD	Mdn	min	max	n	M	SD	Mdn	min	max	n	M	SD	Mdn	min	max
MSCEIT^a^	102	98	10	99	65	118	97	96	12	97	41	117	199	97	11	97	41	118
MCCQE Part II^b^	102	554	64	558	372	677	97	571	57	571	423	689	199	562	61	563	372	689

Abbreviations: MSCEIT = Mayer-Salovey-Caruso Emotional Intelligence Test; MCCQE Part II = Medical Council of Canada Qualifying Examination Part II.

a = Mean score = 100, SD = 15

b = mean examination score = 500, SD = 100.

For the combined cohorts, the relationship between scores can be seen in Figure 2. Participants with low or high MSCEIT scores had a range of MCCQE Part II scores with a correlation of *r*(197) = .06, P =.41, 95% CI [-.08, .20]. Age and gender accounted for some of the variance in MCCQE Part II scores (*F*(2,196) = 5.10, *R*^2^ = .05, *p* = .007) but the addition of the MSCEIT led to a non-significant increase in *R*^2^ of .002 (*F*(1,195)=.36, *R*^2^ = .05, *R*^2^ change = .002 *p* =.55). For 2006, the correlation between the MSCEIT and MCCQE Part II scores was *r*(100) = .09, P = .39, 95% CI [-.11, .28]. The covariates of age and gender did not account for a statistically significant amount of variance (*F*(2,99) = 2.45, *R*^2^ = .05, p=.09). There was a non-significant increase in *R*^2^ of .007 when the MSCEIT V2.0 was considered (*F*(1,98) =.68, *R*^2^ = .05, *p* =.41. For the 2007 cohort, the correlation between scores was *r*(95) = .06, P = .52, 95% CI [-.14, .26]. The covariates of age and gender did account for a significant amount of variance in scores (*F*(2,94) = 2.63, *R*^2^ = .05, *p* =.08) and there was a non-significant increase in *R*^2^ of .00 when the MSCEIT score was considered (*F*(1,93) =.03, *R^2^* = .05, p =.86).

## Discussion

The purpose of this study was to explore the use of EI as a medical school admissions tool to determine if the MSCEIT v2.0 could predict future scores on a national licensure examination. The main finding was that after controlling for the effect of gender and age, the MSCEIT v2.0 scores were not significantly correlated with licensing examination scores. It is perhaps not surprising the MSCEIT v2.0 scores did not correlate with the MCCQE Part I, given that MSCEIT scores are designed to measure aspects of perceiving and managing emotions and the MCCQE Part I is designed to measure general medical knowledge and clinical decision-making skills. It is surprising though that there were non-significant correlations with the MCCQE Part II. Although this examination is not explicitly a measure of non-academic skills, there are aspects of performance related to communication and patient interaction that would be considered non-academic skills and contribute to the examination score. We believed that these aspects wouldhave produced higher correlations between the scores on the MCCQE Part II and the MSCEIT.

Finding no correlation between EI scores and licensure exam scores isconsistent with other studies that have found a poor relationship between EI scores and other measures at admissions during medical school^13^^,^^15^^,^^18^^,^^25^^,^^38^ and on the USMLE Step 1.^33^These findings, however, are in contrast to studies that have found statistically significant correlations between EI scores and scores of other measures during medical school including written tests,^20^ interpersonal skills,^21^ clinical and knowledge-based exams,^23^ and the USMLE Step 2 and 3.^33^

It is possible that the discrepancy between findings in these studies is related to how EI is measured. For some studies, EI is considered a trait and measured with rating scales like TEIQue and the EQi. Other studies consider EI to be a cognitive ability and measure it using ability tests like MSCEIT v2.0 or situational judgment tests like the STEM and the STEU. These rating scales have different assumptions and formats, which might contribute to the discrepant findings between studies. In addition, typically trait EI scores tend to be poorly correlated with the MSCEIT, the STEM and the STEU.^27^ Another possible explanation for the discrepant findings in the literature could be that some medical schools and training programs offer courses related to developing EI skills whereas others do not. In our study, students did not receive training in EI related skills as part of their medical degree. Although MCC would be aware of the training program, given it is a requirement of the MCCQE Part II, we did not ask for this information and therefore we cannot speak to whether EI training versus no EI training would have influenced the correlations.

There are two limitations that could influence the interpretation of our findings. The first is that participation in this study was voluntary, and therefore participants self-selected whether they would participate. This self-selection process runs the risk of recruiting a non-representative sample of students into the study. A second limitation is that only students offered an interview were given a chance to participate in this study. Because admissions procedures vary at each medical school, the screening process used to select students to be interviewed at our medical school could have selected a sample of students that would differ from those selected at another medical school. However, given the range of MSCEIT scores reported in Table 1, it is unlikely that either of these factors is an issue. A third limitation is that this study focused on EI at admissions in order to determine if it could be used as part of the selection process. This study did not investigate if the correlation between the MSCEIT score and the licensing examination change over time. It is possible that as students mature over their training, their EI could change. If so, MSCEIT scores at the end of training may have a different correlation with the licensing examination than a MSCEIT score at the time of admissions. Previous studies have looked at the stability of EI across medical training and found that EI scores remain stable across time.^39^ We have no reason therefore to expect a change in the pattern of our results over time.

One could argue that the population of medical students is not typical of the reference sample which is used for scoring the MSCEIT items and therefore would not be an appropriate measure of EI. This question has been studied by Brannick and colleagues and although there may be issues with some of the MSCEIT sub-scores, their conclusion is that the MSCEIT total score can apply to medical students.^28^^,^^29^ One could also argue that the low correlations found in this study were due to the licensure examination being a poor measure of clinical knowledge and skills rather than attributing the low correlation to the MSCEIT v2.0 as we did. However, MCC examinations are high stakes examinations that are used as the national standard in Canada for assessing the competency of medical school graduates. As such they are created and developed in a rigorous manner and several studies and technical reports have provided validity evidence for the examination results.^41^

In conclusion, an important aspect of validity evidence for any assessment tool is that scores that measure similar constructs should be related. Furthermore, if EI is to be used as an admissions tool, it should predict future performance at least to some degree for related constructs. Our findings, combined with other studies involving this cohort could lead one to question the validity of using EI as a non-academic measure for admissions.^15^^,^^25^ However, it may be possible that EI is measuring something very different from the other admission tools and undergraduate measures of success that were reported in our studies. If so, then our results would be expected and using EI at admissions would ensure unique information about the applicants would be considered. It is not clear which of these interpretations is most accurate given the variation involving both similar and different measures of EI in all of the other studies that have compared EI to other medical school measures. At the very least, one should be cautious if considering using a measure of EI as part of the admissions process. We recommend ongoing research, such as exploring the potential relationship between EI and future regulatory complaints or investigating why there is such variation across the different studies that have compared EI to measures of success in medical school.
